# Prophylactic surgical drainage is associated with increased infection following intramedullary nailing of diaphyseal long bone fractures: A prospective cohort study in Nigeria

**DOI:** 10.1051/sicotj/2020003

**Published:** 2020-02-18

**Authors:** Gerald Chukwuemeka Oguzie, Patrick Albright, Syed Haider Ali, Ndubuisi E. Duru, Emmanuel Chino Iyidobi, Omolade Ayoola Lasebikan, Denning C. Chukwumam, Hao-Hua Wu, Ikpeme A. Ikpeme

**Affiliations:** 1 Consultant Orthopaedic & Trauma Surgeon, Federal Medical Center Orlu Road Owerri Imo State Nigeria; 2 Institute for Global Orthopaedics and Traumatology, University of California, San Francisco 2550 23rd Street, Building 9, 3rd Floor San Francisco CA 94110 USA; 3 Consultant Orthopaedic Surgeon, National Orthopaedic Hospital, Enugu Abakpa junction Abakiliki Express Road Enugu P.M.B. 01294 Enugu State Nigeria; 4 Consultant Orthopaedic Surgeon, Federal Medical Center Orlu Road Owerri Imo State Nigeria; 5 Consultant Orthopaedic Surgeon, University of Calabar Teaching Hospital Court Rd Duke Town, Calabar Cross River State Nigeria

**Keywords:** Surgical drain, Nigeria, femur fracture, tibia fracture, intramedullary nail

## Abstract

*Introduction*: Prophylactic surgical drains are commonly used in Nigeria following intramedullary nailing (IMN) of long bone diaphyseal fractures. However, evidence in the literature suggests that drains do not confer any benefit and predispose clean wounds to infection. This study compares outcomes between patients treated with and without prophylactic surgical drainage following diaphyseal long bone fractures treated with IMN. *Methods*: A prospective cohort study with randomization was conducted at a tertiary referral center in Enugu, Nigeria. Investigators included skeletally mature patients with diaphyseal long bone (femur, tibia, humerus) fractures treated with SIGN IMN. Patients followed-up at 5, 14, and 30 days post-operatively. The primary outcome was surgical site infection (SSI) rate. Secondary outcomes included post-operative pain at 6 and 12 h, need for blood transfusion, wound characteristics (swelling, ecchymosis, and gaping), need for dressing changes, and length of hospital stay. *Results*: Of the enrolled patients, 76 (96%) of 79 completed 30-day follow-up. SSI rate was associated with patients who received a prophylactic drain versus those who did not (23.7% vs. 10.5%, *p* = 0.007). There were no significant differences in transfusion need (*p* = 0.22), wound swelling (*p* = 0.74), wound ecchymosis (*p* = 1.00), wound gaping (*p* = 1.00), dressing change need (*p* = 0.31), post-operative pain at 6 h (*p* = 0.25) or 12 h (*p* = 0.57), or length of stay (*p* = 0.95). *Discussion*: Surgical drain placement following IMN of diaphyseal long bone fractures is associated with a significantly higher risk of SSI. Reducing surgical drain use following orthopaedic injuries in lower resource settings may translate to reduced infection rates.

## Introduction

Prophylactic surgical wound drains are commonly used in resource poor settings, particularly following intramedullary nailing (IMN) of long bone diaphyseal fractures with readily available Surgical Implant Generation Network (SIGN) nails [1–3]. Drains are used in these settings due to the lack of advanced operative equipment such as electrocautery and minimal access techniques, and it continues to remain the standard of care in some resource limited settings [4]. Prophylactic surgical drainage following musculoskeletal injuries has been suggested to reduce hematoma formation and surgical site infection (SSI), to decrease wound swelling and the sequelae of compartment syndrome, and to enhance the local wound environment [5–8].

However, the indications for drain use in resource-poor settings are controversial. Recent evidence suggests that prophylactic drains do not confer any benefit and predispose clean wounds to infection [4, 9]. Wound drainage following open reduction and internal fixation of femoral fractures has shown no significant difference in the rate of wound infection [6, 10–12]. Thus, prophylactic surgical drainage following clean IMN procedures occurs in the absence of evidence demonstrating improved patient outcomes and contributes to increased cost of care. Ikpeme et al. reported that prophylactic wound drainage conferred no patient outcome advantages while contributing to increased patient costs in a Nigerian teaching hospital [4].

There is limited evidence in the literature describing the use of surgical drains for wound management in resource limited settings, particularly as it pertains to orthopaedic surgeries. The additional cost to patients and the unresolved effect of drains on patient outcomes necessitates further study of prophylactic drainage in orthopaedic surgical procedures in resource poor settings [6–8, 13]. This study aims to fill this gap in the literature by determining patient outcomes following prophylactic surgical wound drainage versus no-drainage, among patients with diaphyseal long bone fractures treated with SIGN IMN.

## Materials and methods

We conducted a prospective cohort study with randomization at a tertiary referral center in Enugu, Nigeria between January 2016 and December 2016. Institutional review board approval was obtained from the hospital ethical committee. Informed consent was obtained from all individual participants included in the study. Sample size was calculated as 79 patients based on data collected from a pilot study, with *α* (two-tailed) set at 0.05 and *β* at 0.20.

All adult patients presenting to the Outpatient Surgery or Accident and Emergency departments with closed, extraarticular diaphyseal femoral, tibial, or humeral fractures treated with the SIGN IMN (SIGN Fracture Care International, Richland, WA) were screened. Patients were enrolled if they provided written informed consent and did not meet any of the following exclusion criteria: (a) delayed presentation (> 3 months first contact post-fracture); (b) open fracture; (c) malunion or non-union; (d) intra-articular fractures; (e) skeletal immaturity; or (f) history of immunosuppression. Immunosuppression history was defined as either positive history of HIV/AIDS, poorly controlled diabetes mellitus (fasting blood glucose ≥ 7.1 mmol/L at pre-operative evaluation), or prior extended use of systemic steroids.

Patients were randomized to each treatment arm in the pre-operative setting using sequential randomization. The study team cut paper into pieces with drain or no drain written on each piece and selected these pieces from an envelope. Information recorded at enrollment included patient demographics, comorbidities, injury factors, and treatment factors. The control group received a closed suction drain at the time of wound closure per standard of care at the study setting. The intervention group received no suction drain at the time of wound closure. The surgeon performing the procedure was blinded to the patient’s trial arm until the time of wound closure when the drain was placed.

All patients received pre-operative systemic antibiotics (1-gram IV Ceftriaxone). Consultant surgeons or senior registrars under the direct supervision of a consultant performed all surgeries and used standard surgical approaches for each long bone diaphyseal fracture [14]. All cases were completed utilizing an open reduction procedure in order to assist with fracture reduction and subsequent placement of the SIGN IMN in either an antegrade or retrograde manner. All intervention group patients received a sterile 50 mm closed Redivac OEC 1800 HS drain tube (Northamptonshire, UK) attached to a negative pressure suction bottle at the time of wound closure. The surgical team inserted drains at the wound site for the fracture reduction and anchored them under the fascia layer with a non-absorbable suture. They applied sterile gauze and cotton-wool dressings to the wound and around the drain site.

Nursing staff, not otherwise involved in the study, removed drains 48 h after insertion in a standardized manner without disruption of the wound site. Nursing staff additionally assessed patient pain at 6 h and 12 h post-operatively via 10-point Visual Analog Scale (VAS).

A rotating group of surgeons evaluated patients on post-operative days 5 (inpatient), 14 (outpatient visit, time of suture removal), and 30 (outpatient visit). The primary outcome of the study was surgical site infection (SSI). Study personnel evaluated wounds for infection using Centers for Disease Control and Prevention (CDC) criteria [15] and assessed wound ecchymosis and wound swelling on post-operative day 5. Ecchymosis was defined as appreciable hyperpigmentation within 3 cm of wound borders and swelling was a clinical diagnosis defined as appreciable swelling at the wound site. The study group also evaluated wound gaping on post-operative day 14 following suture removal and defined it as poor approximation of wound edges following suture removal with exposure of deep tissue.

Descriptive and analytic statistics were performed using STATA (StataCorp LLC, College Station, TX). Continuous data were assessed via Student’s *t*-test for parametric data and the Wilcoxon rank-sum test for non-parametric data. Categorical data were assessed via Pearson’s Chi-square test and Fisher’s exact test. A multivariate logistic regression was performed to assess the influence of baseline characteristics on risk of SSI.

## Results

**Figure 1:**
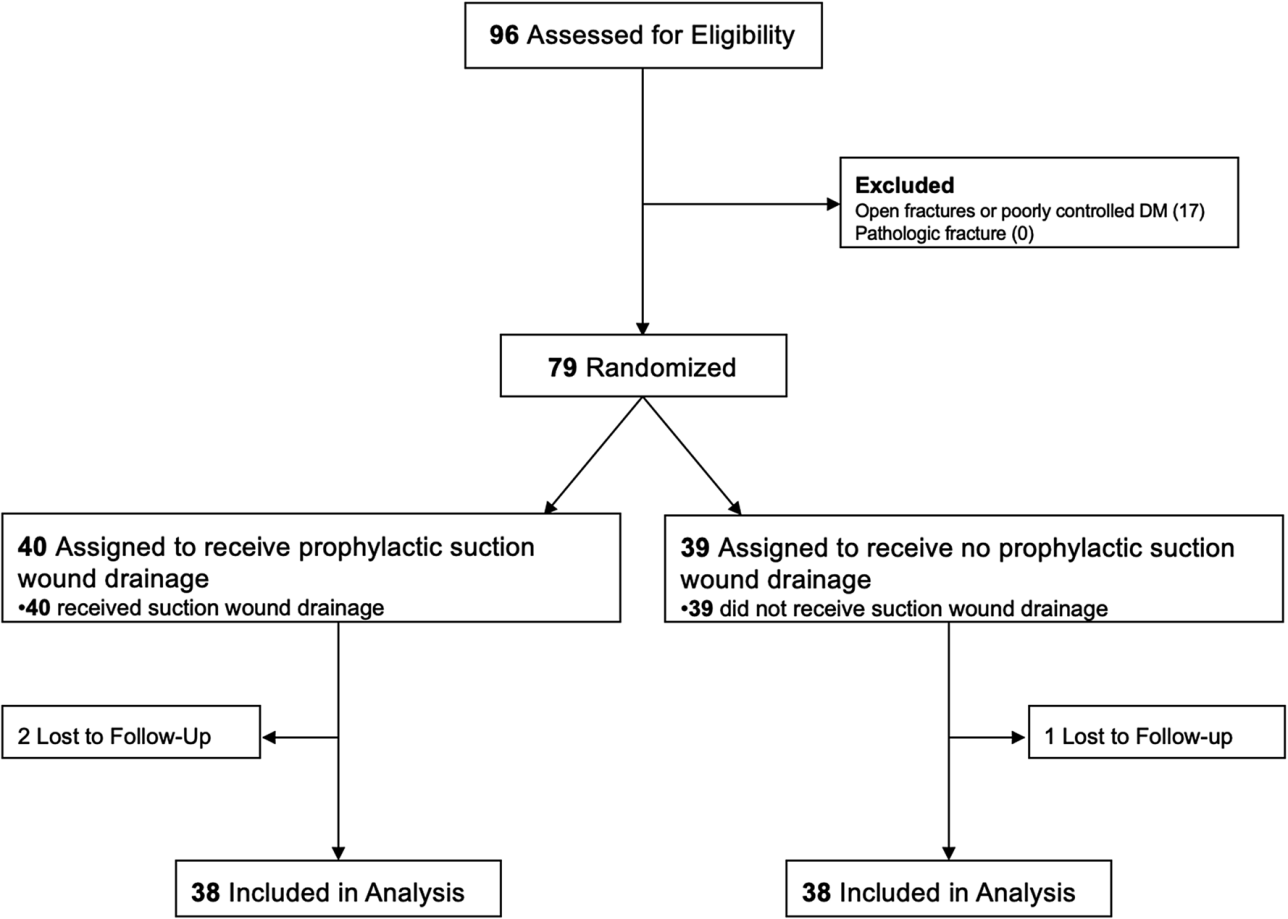
CONSORT flow diagram illustrating screening, enrollment, randomization and follow-up.

A total of 96 patients with extraarticular diaphyseal long bone fractures treated with SIGN IMN were screened, and 79 were enrolled (Figure 1). Three patients were lost to follow-up following discharge from the hospital (1 in the control group, and 2 in the intervention group). The remaining patients completed a 30-day follow-up and were included in data analysis.

There were 38 patients in each study group. There were no significance differences between the control and intervention groups with regard to age, sex, mechanism of injury, injury laterality, anatomic site of the fracture along the diaphysis, or by surgeon training status (Table 1). However, patients in the drain group were more likely to have experienced humeral fractures, whereas patients in the no drain group were more likely to have experienced femoral fractures (*p* = 0.002).

**Table 1:** Patient demographics and peri-operative characteristics.

Category	Subcategory	Drain		No drain		*p*-value
		*N* = 38	(%)	*N* = 38	(%)	
Age	Mean (*SD*)	40.5	(16.9)	36.2	(12.4)	0.21
Sex	Male	23	(61)	29	(76)	0.14
	Female	15	(39)	9	(24)	
Patient occupation	Trading	7	(18)	8	(21)	0.073
	Housewife	4	(11)	2	(5)	
	Student	8	(21)	5	(13)	
	Commercial cyclist	0	(0)	7	(18)	
	Corps member	4	(11)	2	(5)	
	Engineer	6	(16)	1	(3)	
	Clergy	3	(8)	2	(5)	
	Teaching	3	(8)	5	(13)	
	Driver	0	(0)	3	(8)	
	Artisan/electrician	0	(0)	1	(3)	
	Civil servant	2	(5)	1	(3)	
	Medical doctor	1	(3)	0	(0)	
	Farming	0	(0)	1	(3)	
Mechanism of injury	Motor vehicle	15	(39)	15	(39)	0.94
	Motorcycle	8	(21)	11	(29)	
	Tricycle	6	(16)	5	(13)	
	Pedestrian	2	(5)	3	(8)	
	Fall from height	2	(5)	1	(3)	
	Sports	1	(3)	0	(0)	
	Fall on slippery ground/floor	3	(8)	2	(5)	
	Object fell onto thigh	1	(3)	1	(3)	
Injury laterality	Right	19	(50)	20	(53)	0.38
	Left	15	(39)	17	(45)	
	Both	4	(11)	1	(3)	
Bone involved	Femur	9	(24)	23	(61)	0.002
	Tibia	13	(34)	10	(26)	
	Humerus	16	(42)	5	(13)	
Site of fracture	Proximal diaphyseal	15	(39)	11	(29)	0.48
	Middle diaphyseal	19	(50)	20	(53)	
	Distal diaphyseal	4	(11)	7	(18)	
EBL	Mean (*SD*)	207.4	(132.9)	246.1	(132.2)	0.21
Surgeon training level	Consultant	23	(61)	20	(53)	0.49
	Senior registrars	15	(39)	18	(47)	

At final follow-up, 11 patients developed SSI; 10 in the drain group and 1 in the no drain group (Table 2, *p* = 0.007). There were no significant differences between the drain and no drain groups with regards to blood transfusion requirements during the post-operative period (*p* = 0.222), wound swelling (*p* = 0.736), wound ecchymosis (*p* = 1.000), wound gaping (*p* = 1.000), or need for dressing changes (*p* = 0.305). Similarly, there were no significance differences between the control and intervention groups with regards to post-operative pain at 6 h (*p* = 0.249), 12 h (*p* = 0.566), or in length of hospital stay (*p* = 0.949).

**Table 2:** Primary and secondary outcomes by prophylactic surgical drain vs. no drain.

	Drain	No drain	Risk difference	*RR*	*p*-value*
	Risk	Risk			
Surgical site infections	0.263	0.026	−0.237	0.1	0.007
Post-operative blood transfusion	0.237	0.105	−0.132	0.444	0.222
Wound characteristics					
Swelling	0.105	0.158	0.053	1.5	0.736
Ecchymosis	0.132	0.158	0.026	1.2	1.000
Gaping	0	0.026	0.026	1.0	1.000
Dressing change	0.211	0.342	0.132	1.625	0.305
	Mean	Mean	∆		*p*-value
Post-operative pain					
6 h	5.842	6.211	0.368		0.249
12 h	3.105	3.289	0.184		0.566
Length of hospital stay (days)	11.263	11.158	−0.105		0.949

* Using chi-square and student *t*-test *p* < 0.05.

On multivariate analysis, absence of a prophylactic drain was associated with decreased odds of SSI (Table 3, OR = 0.042, *p* = 0.015). The bone involved was not associated with SSI in the model (*p* = 0.531). Both patient gender (female > male, *p* = 0.036) and surgeon status (senior registrar > consultant, *p* = 0.025) were associated with SSI.

**Table 3:** Multivariate logistic regression model of baseline covariates associated with surgical site infections.

	Odds ratio	95% Confidence interval	Standard error	*p*-value
Absence of drain	0.042	0.003–0.54	0.055	0.015
Bone fractured	1.723	0.31–9.46	1.497	0.531
Sex	7.249	1.14–46.26	6.855	0.036
Surgeon status	7.942	1.30–48.50	7.332	0.025
Cumulative				0.0011

Overall *p*-value = 0.0011 and pseudo-*R* ^2^ = 0.3304 (significance set at *p* < 0.05).

## Discussion

The aim of this prospective cohort study with randomization was to compare outcomes between patients treated and not treated with prophylactic surgical drainage following SIGN IMN of diaphyseal long bone fractures. The primary outcome was rate of SSI. This is the first prospective study in a resource limited setting to present outcomes of prophylactic drain use in patients with traumatic long bone fractures, and this study is additionally relevant to surgeons in resource limited settings where drains continue to be the standard of practice for the post-operative management of long-bone fractures. We find that not using drains is associated with a significant reduction of SSI following IMN of long bone fractures.

Despite randomization, the control group contained more humerus fractures, whereas the intervention group contained more femoral fractures at the time of enrollment. To control for the effect of bone type, we conducted a multivariate logistic regression of drain use, bone type, sex and surgeon status against SSI. Bone type was, perhaps surprisingly, not associated with SSI on multivariate analysis. There is evidence that infections are generally less common in upper extremity fractures as compared to lower extremity traumatic fractures [16, 17]. The data for this variable in our study demonstrate a wide confidence interval, and our study is likely underpowered to detect a difference beyond that of differing infection rates among the entire patient cohort. In patients with SSI, all infections were superficial and responded to culture sensitive antibiotic therapy and local wound care. These findings are consistent with results from a study of orthopaedic surgery patients of any type that reported a 12.8% rate of superficial wound infection with drain use and 3.2% without drain use [4]. Our findings support the evidence that drain use in orthopaedic wounds is associated with a higher risk of surgical site infection [4, 18]. However, prior literature is equivocal with several studies demonstrating no difference or even decreased rates of infection with drain use [10, 19, 20]. Multi-center studies with standardized drain procedures would clarify the effect of prophylactic drain use on the infection rate in orthopaedic surgical wounds.

Interestingly, females demonstrated a strong association with increased risk of SSI compared to males. Some previous Nigerian literature has demonstrated a trend towards increased infections in females following surgical procedures of any type [18, 21–23]. Conversely, other Nigerian studies have demonstrated no significant difference between sexes in risk of SSI following orthopaedic procedures [24–26]. Local cultural factors may be the source of this difference. However, it should be noted that there were discrepant proportions among the treatment arms between genders, and there were more females in the control group that ultimately had more SSI. There is a need for future studies to establish whether this is a true finding.

We additionally report that lower level of surgical training is associated with risk of SSI. Surgeries conducted by a senior registrar under the supervision of a consultant yielded a higher rate of SSI in patients receiving drains. Senior registrars in Nigeria are surgeons who have completed their primary training but are not permitted to perform independent operation. They are comparable to fellows in the United States. The increased risk of infection associated with senior registrars may be due to a multitude of factors including lack of experience in managing complex surgical wounds, increased operation duration, and decreased operative volume [27, 28]. Increased trainee participation has been associated with increased risk of SSI in the orthopaedic literature [29], and it is important for consultants to ensure that trainees are provided adequate assistance in minimizing operation duration and treating complex orthopaedic pathology.

The results of this study may also have cost implications regarding drain use. Lowering healthcare associated costs is an ongoing objective of many low- and middle-income countries (LMICs), and the use of drains in orthopaedic surgery is associated with increased cost [4, 30]. Khanal et al. reported that cost of treatment, inclusive of drain use, was higher than other routine orthopaedic cases [19], and the average drain costs in similar studies have been cited as 25–30 USD [4, 10]. Although drain cost is variable, it adds to the financial burden of healthcare for many patients in LMICs.

This study is novel in that it is exceedingly difficult to conduct an effective prospective cohort study with randomization in a LMIC. However, there are limitations. There is a disparity in the distribution of fractures by bone between the control and the intervention groups. Given that other baseline characteristics were similar between groups, this disparity is likely a product of small sample size. The study is also underpowered to detect a difference for various subgroup analyses of the individual long bones. Assessment of wound gaping, swelling, and ecchymosis is subjective, and although the same group of 3 surgeons assessed each wound, we did not assess intra-observer reliability. The follow-up period duration in this study was limited to one month, because we hypothesized that the impact of drain use on SSI would fall in the immediate post-operative period. The follow-up duration led to a 96% follow-up rate which is a significant achievement in the study setting. Future studies would ideally follow-up patients over the entire 1-year follow-up period. Finally, we report data obtained from a single institution in Nigeria which raises concerns of generalizability to other centers in different resource limited settings.

## Conclusion

We report novel data from a prospective cohort study with randomization in a resource limited country with significant implications for management of orthopaedic patients. We find that surgical drain use following SIGN nailing of diaphyseal long bone fractures is associated with a significantly higher risk of surgical site infection. Reduced use of surgical drains following orthopaedic injuries in lower resource settings may translate to lower rates of infection.

## Ethical approval

All procedures performed in studies involving human participants were in accordance with the ethical standards of the institutional and/or national research committee and with the 1964 Helsinki declaration and its later amendments or comparable ethical standards.

## Conflict of interest

On behalf of all authors, the corresponding author states that there is no conflict of interest.

## Funding

There were no external funding sources for this study.

## References

[1] Soren OO , Moi M , Makerere MO , Surgeon CO , Provincial E (2009) Outcome of surgical implant generation network nail initiative in treatment of long bone shaft fractures in Kenya. E African Ortho J 3(1), 7–14.

[2] Hassan W , Atiq G , Hassan M (2013) Treatment of nonunion of long bone fractures with surgical implant generation network nail. J Surg Pakistan 18(2), 64–67.

[3] Ikpeme I , Ngim N , Udosen A , Onuba O , Enembe O , Bello S (2011) External jig-aided intramedullary interlocking nailing of diaphyseal fractures: experience from a tropical developing centre. Int Orthop 35, 107–111. 20148329 10.1007/s00264-009-0949-0 PMC3014482

[4] Ikpeme IA , Ngim NE , Ilori IU , Oku E , Udosen AM (2013) Prophylactic wound drainage in orthopaedics: a comparative evaluation of closed suction drainage versus no-drainage in a nigerian teaching hospital. Surg Sci 4, 277–282.

[5] Smith SG , Shapiro MS (1997) The use of drains for outpatient orthopaedic surgeries: Safety and efficacy. Ambul Surg 5(4), 145–147.

[6] Cheung K , Chiu K (2006) Effect of drain pressure in total knee arthroplasty. J Orthop Surg 14(2), 163–166. 10.1177/230949900601400211 16914781

[7] El Khalifa T , Al Mahozi A , Dhaif B (2008) Intra-articular drain versus no drain after arthroscopic anterior cruciate ligament reconstruction: A randomized prospective clinical trial. *Bahrain Med Bull* 30(1). 10.1016/j.arthro.2006.05.004 16904589

[8] Berman AT , Fabiano D , Bosacco SJ , Weiss AA (1990) Comparison between intermittent (spring-loaded) and continuous closed suction drainage of orthopedic wounds: a controlled clinical trial. Orthopedics 13(3), 309–314. 2179912 10.3928/0147-7447-19900301-10

[9] Gaines RJ , Dunbar RP (2008) The use of surgical drains in orthopedics. Orthopedics 31(7), 702–705. 18705564 10.3928/01477447-20110505-06

[10] Lawal YZ , Ogirima MO , Dahiru IL , Abubakar K , Ajibade A (2014) On the use of drains in orthopedic and trauma. Niger J Clin Pract 17(3), 366–369. 24714019 10.4103/1119-3077.130246

[11] Akinyoola AL , Odunsi A , Yusuf MB (2012) Use of wound drains following open reduction and internal fixation of femoral shaft fractures. J Wound Care 21(6), 279–284. 22886293 10.12968/jowc.2012.21.6.279

[12] Chandratreya A , Giannikas K , Livesley P (1998) To drain or not drain: literature versus practice. J R Coll Surg Edinb 43(6), 404–406. 9990789

[13] Parker MJ , Livingstone V , Clifton R , McKee A (2007) Closed suction surgical wound drainage after orthopaedic surgery. Cochrane Database Syst Rev 3, CD001825. 10.1002/14651858.CD001825.pub2 PMC8408575 17636687

[14] Zirkle LG (2011) Technique Manual of SIGN IM Nail & Interlocking Screw System Insertion & Extraction Guide. Richland, WA: Surgical Implant Generation Network, 37 p.

[15] Garner JS (1986) CDC guideline for prevention of surgical wound infections, 1985. Supersedes guideline for prevention of surgical wound infections published in 1982. Revised. *Infect Control* 7(3), 193–200. 3633903 10.1017/s0195941700064080

[16] Dellinger EP , Miller SD , Wertz MJ , Grypma M , Droppert B , Anderson PA (1988) Risk of infection after open fracture of the arm or leg. Arch Surg 123(11), 1320–1327. 3178479 10.1001/archsurg.1988.01400350034004

[17] Fernandes MC , Peres LR , Queiroz Neto AC , Lima Neto JQ , Turibio FM , Matsumoto MH (2015) Open fractures and the incidence of infection in the surgical debridement 6 hours after trauma. Acta Ortop Bras 23(1), 38–42. 26327794 10.1590/1413-78522015230100932 PMC4544519

[18] Ikeanyi UOE , Chukwuka CN , Chukwuanukwu TOG (2013) Risk factors for surgical site infections following clean orthopaedic operations. Niger J Clin Pract. 16(4), 443–447. 23974736 10.4103/1119-3077.116886

[19] Khanal G , Rijal R , Shrestha B , Karn N , Chaudhary P (2011) A study to evaluate the role of suction drains in orthopedic surgery. Health Renaiss 9(2), 91–94.

[20] Tjeenk RM , Peeters MPV , van den Ende E , Kastelein GW , Breslau PJ (2005) Wound drainage versus non-drainage for proximal femoral fractures. Injury 36(1), 100–104. 15589927 10.1016/j.injury.2004.04.011

[21] Olowo-Okere A , Ibrahim YKE , Sani AS , Atata RF , Olayinka BO (2017) Prevalence of surgical site infection in a Nigerian university teaching hospital. J Pharm Allied Sci 14(1), 2430–2438.

[22] Olowo-okere A , Ibrahim YKE , Sani AS , Olayinka BO (2018) Occurrence of surgical site infections at a tertiary healthcare facility in Abuja, Nigeria: A prospective observational study. Med Sci 6(3), 60. 10.3390/medsci6030060 PMC6163208 30061516

[23] Thahir M , Gandhi S , Kanniyan K , Kumar R , Thahir M , Orthop JR (2018) A prospective study of surgical site infection of orthopedic implant surgeries. Int J Res Orthop 4(1), 1–7.

[24] Amoran O (2014) Rates and risk factors associated with surgical site infections in a tertiary care center in south-western Nigeria. Int J Trop Dis Heal 3(1), 25–36.

[25] Kimmatkar N , Hemnani J (2017) Incidence of surgical site infections in IPD Orthopedics patients undergoing implant surgery. Int Arch Biomed Clin Res 3(4), 135–138.

[26] Langelotz C , Mueller-Rau C , Terziyski S , et al. (2014) Gender-Specific Differences in Surgical Site Infections: An Analysis of 438,050 Surgical Procedures from the German National Nosocomial Infections Surveillance System. Viszeralmedizin 30(2), 114–117. 26288585 10.1159/000362100 PMC4513817

[27] Uçkay I , Hoffmeyer P , Lew D , Pittet D (2013) Prevention of surgical site infections in orthopaedic surgery and bone trauma: state-of-the-art update. J Hosp Infect 84(1), 5–12. 23414705 10.1016/j.jhin.2012.12.014

[28] Waltz PK , Zuckerbraun BS (2017) Surgical site infections and associated operative characteristics. Surg Infect (Larchmt) 18(4), 447–450. 28448197 10.1089/sur.2017.062

[29] Olsen MA , Nepple JJ , Riew KD , et al. (2008) Risk factors for surgical site infection following orthopaedic spinal operations. JBJS 90(1). 10.2106/JBJS.F.01515 18171958

[30] Bjerke-Kroll BT , Sculco PK , McLawhorn AS , Christ AB , Gladnick BP , Mayman DJ (2014) The increased total cost associated with post-operative drains in total hip and knee arthroplasty. J Arthroplasty 29(5), 895–899. 24360337 10.1016/j.arth.2013.10.027

